# Homosalate and ERK Knockdown in the Modulation of *Aurelia coerulea* Metamorphosis by Regulating the PI3K Pathway and ERK Pathway

**DOI:** 10.3390/cimb46100690

**Published:** 2024-10-18

**Authors:** Jinhong Chen, Xiaoyu Geng, Bingbing Li, Jinyao Xie, Jieying Ma, Zhen Qin, Mingke Wang, Jishun Yang

**Affiliations:** Naval Medical Center of PLA, Naval Medical University, Shanghai 200052, China; 15618421309@163.com (J.C.); 15339625597@163.com (X.G.); m18131452435@163.com (B.L.); xiexie192471@163.com (J.X.); qinzhen1990gfs@126.com (Z.Q.)

**Keywords:** homosalate, *Aurelia coerulea*, metamorphosis, PI3K pathway, ERK pathway

## Abstract

Metamorphosis control is pivotal in preventing the outbreak of jellyfish, and it is often studied using common model organisms. The widespread use of the ultraviolet blocking agent homosalate in cosmetics poses a threat to marine ecosystems. Although the impact of homosalate on marine organisms has been extensively examined, there is a notable absence of research on its effects on jellyfish metamorphosis and the underlying mechanisms, warranting further investigation. In this study, we first established a study model by using 5-methoxy-2-methylindole to induce *Aurelia coerulea* metamorphosis, and selected homosalate as a PI3K agonist and an ERK agonist, while we used YS-49 as a specific PI3K agonist, as well as ERK knockdown, to observe their effect on the metamorphosis of *Aurelia coerulea*. The results showed that an *Aurelia coerulea* metamorphosis model was established successfully, and the PI3K agonist homosalate, YS-49, and the knockdown of ERK molecules could significantly delay the metamorphosis development of *Aurelia coerulea*. We propose that activating PI3K/Akt and inhibiting the ERK pathway are involved in the delayed development of *Aurelia coerulea*, which provides a new strategy for the prevention and control of jellyfish blooms.

## 1. Introduction

In recent years, jellyfish outbreaks have occurred frequently due to climate warming, environmental pollution, overfishing, and other reasons [1]. There are approximately 1000 species of jellyfish in the world [2]. Jellyfish stings cause serious marine biological injuries and are a critical public health concern. There are about 150 million jellyfish stings worldwide each year, with up to 800 daily [3,4]. Thousands of people in the Pacific coastal areas die of jellyfish stings [5]. At least 63 people have died from jellyfish stings in the subtropical waters of Australia [6], and 20–50 people die annually from jellyfish stings in coastal areas of Thailand and the Philippines [7,8]. The common symptoms of jellyfish stings include itching, skin swelling, and severe organ failure, resulting in rapid death [9,10,11,12].

The jellyfish is a mysterious and ancient drifting creature that belongs to the phylum Cnidaria. Jellyfish have lived in the ocean for more than 600 million years [13], and are distributed worldwide [14,15,16,17,18,19]. Jellyfish have been studied for nearly 150 years and are used as models in many domains, including systematic and evolutionary neuroscience [20,21], evolutionary science [22], kinetics [23,24], ototoxicity [25], regeneration [26,27], jellyfish poisoning syndrome [28,29], drug administration [30], and marine watershed environmental monitoring [31]. Large-scale jellyfish blooms in the coastal waters of China mainly include *Aurelia aurita*, *cyanea capillate*, and *Nemopilema nomura* [32]. *Aurelia aurita* (sea-moon jellyfish) belongs to Cnidaria, Scyphomedusae, Semaeostomae, and Aurelia, which are distributed globally. Although the lethality of *Aurelia coerulea* stings has rarely been reported, skin, cardiac, hepatic, and renal injuries caused by its venom have been reported in previous studies, and their commensal microorganisms may cause human infections [33,34,35,36]. The asexual reproduction and strobilation of the sea-moon jellyfish directly determine the number of jellyfish disks and thus determine the population dynamics and characteristics of the jellyfish. Polyps can produce several or even dozens of nodal disks via strobilation, each node disk can fall off, and the disk body can further develop into a jellyfish body. Therefore, the process of abnormal development plays a crucial role in jellyfish blooms [37].

Metamorphosis is a basic life feature of jellyfish [38,39]. The metamorphic development cycle of jellyfish encompasses distinct stages: polyp, early strobila, middle strobila, late strobila, and ephyra, culminating in the mature jellyfish stage [40]. When environmental changes such as a drop in temperature occur, fixed polyps will produce strobilas consisting of several to more than a dozen disks, which will become an ephyra and eventually develop into a jellyfish/medusa [39]. Since early strobilation is the starting point of jellyfish metamorphosis, and the number of disks directly depends on the number of ephyra, it is the key process to jellyfish development. Inhibiting the occurrence of early strobila, shortening the metamorphosis cycle, and reducing the number of disks released are important strategies for controlling jellyfish populations [22,41].

There are many signaling pathways involved in regulating the growth and proliferation of hydra, such as the Wnt/β-Catenin signaling pathway, MAPK signaling pathway, PI3K signaling pathway, Hippo signaling pathway, JNK signaling pathway, ERK signaling pathway, Notch signaling pathway, and bone morphogenetic protein (BMP) signaling pathway, which play important roles in the proliferation, regeneration, and bud development processes [42,43,44,45,46]. Numerous studies have shown that the MAPK and PI3K signaling pathways may be related to growth and development [47,48,49], and even directly regulate metamorphosis [50,51]. The results of KEGG data analysis and transcriptome data show that the MAPK and PI3K/Akt signaling pathways do exist in *Aurelia coerulea*, and MAPK and PI3K signaling pathway inhibitors in jellyfish can induce the transformation of the cell cycle [52,53]. However, there are few reports on how signaling pathway molecules regulate the process of metamorphosis in *Aurelia coerulea*. Homosalate is a kind of ultraviolet (UV)-blocking agent [54], which can absorb the UVB 295~315 band and is widely used in cosmetics, household products, personal protective products, and food packaging [54]. Due to its broad applications, it has become the focus of environmental science and industrial research. There is no doubt that it protects skin from UV damage. However, the environmental and social problems caused by its widespread use have become increasingly prominent. With its mass daily consumption comes consistent input into marine ecosystems, directly or indirectly, increasingly threatening them [55]. Homosalate has been found in the tissues of various mammals [56,57]. It can lead to cytotoxicity and DNA oxidative damage [58], endocrine disruption [59,60], skin sensitivity, endometriosis, polycystic ovary syndrome, and reproductive issues [61]. The widespread use of homosalate has also impacted other oceanic species, such as oysters [62], microalgae, Artemia [63], and corals, for example, affecting the growth and reproduction of marine sea urchins and fish [64,65]. However, its effect on jellyfish metamorphosis has not been investigated. Homosalate is not only a PI3K agonist but also an ERK agonist, while YS-49 is a specific PI3K agonist [66,67]. The main purpose of this study was to assess the effects and mechanism of homosalate, the PI3K/Akt signaling pathway, and the ERK pathway on the metamorphosis of *Aurelia coerulea*, so as to provide potential strategies for the prevention and control of jellyfish blooms.

## 2. Materials and Methods

### 2.1. Regents and Animals

Chemically synthesized siRNAs and a riboFECT™CP Transfection Kit were purchased from Ruibo Company (Ribobio, Guangzhou, China). Homosalate and YS-49 were obtained from TargetMol (in Boston, MA, USA). A. coerulea polyps, which were from a subspecies of the genus Aurelia (*Aurelia* sp.), were currently being cultured in our laboratory [68].

### 2.2. Culture of the Polyps and Induction of the Metamorphosis

In our feeding system, the polyps were placed in 10 cm Petri dishes with a feeding density of 20–30 pieces per dish, and the salinity of artificial seawater was controlled in the range of 28‰~32‰. After fresh Fengnian shrimp eggs (Artemia) were prepared and hatched, the food was scattered evenly next to the tentacles of polyps every 2 days. After feeding for 2 h, the water was changed, newly prepared artificial seawater was added, and the mixture was stored in a 20 °C biochemical incubator; the protocol of the feeding method is attached in Appendix A. Pasteur pipettes were used to scrape polyps down along the lines of the corrugated board and put them in a beaker with a volume of 500 mL, then 300 mL artificial seawater with a salinity of 2.8‰–3.2‰ was added into the beaker. It was gently stirred clockwise with a glass rod, then we waited for 1–2 min until impurities formed and the polyps were layered, poured out the impurities on the upper layer, washed the polyps with artificial seawater, and repeated the above steps until the polyps were free from impurities. Then, the polyps could be used in the research.

**Figure 1:**
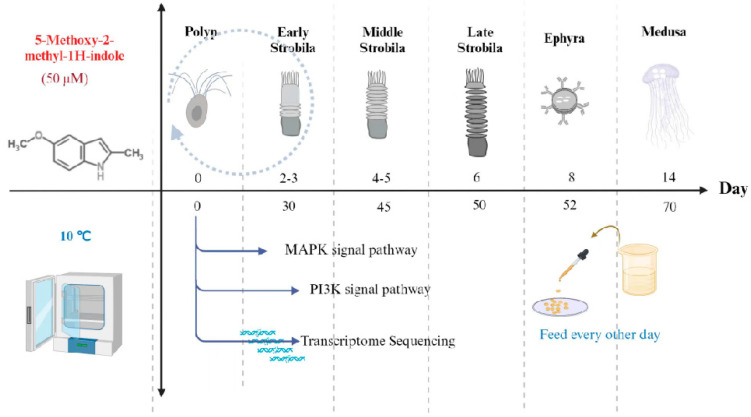
Metamorphosis of *Aurelia coerulea* polyps induced by 5–methoxy–2–methylindole and low temperature.

In order to establish a study model of *Aurelia coerulea* metamorphosis, 0.0081 g of 5-methoxy-2-methylidine (g) was added to 1000 mL of artificial seawater and shaken fully, and then 50 μM 5-methoxy-2-methylidine solution was prepared to induce the metamorphosis of *A. coerulea* polyps. After several budless polyps prepared in advance were put into 6-well plates individually, and 2 mL artificial seawater (control group) and 2 mL different inducers (treatment group) were added to each well, observations were made every 24 h and the results were recorded under a microscope [69]. The process of the metamorphosis of polyps is shown in Figure 1.

### 2.3. Effects of Signal Pathway Agonists Homosalate and YS-49 on the Metamorphosis of Aurelia coerulea

The polyps with good growth conditions were selected from the tank and inoculated into two new 12-well plates two weeks in advance. One week after inoculation, the inoculation was observed, and the polyps could be fed properly (to ensure their health). The budless and hypertrophic polyps were selected, 10 in each well, and one 12-well plate was divided into 3 groups: the 5-methoxy-2-methylindole group, 5-methoxy-2-methylindole + 100 nM homosalate group, and 5-methoxy-2-methylindole + 1 μM homosalate group. The other one consisted of a 5-methoxy-2-methylindole group, 5-methoxy-2-methylindole + 1 μM YS-49 group, 5-methoxy-2-methylindole + 3 μM YS-49 group, 5-methoxy-2-methylindole + 10 μM YS-49 group, 5-methoxy-2-methylindole + 30 μM YS-49 group, and 5-methoxy-2-methylindole + 50 μM YS-49 group. Meanwhile, the corresponding solution (2 mL was added to each well) was cultured in a constant-temperature incubator at 20 °C. The polyps were observed every 24 h and photographed by a stereomicroscope to check. Whether the polyps had strobilated/the time/the number of individuals who strobilated were observed, and the strobilation rate and other indexes were calculated. The strobilation rate was defined as the ratio of the number of strobilations against the number of all samples (including the polyps, strobilations, and ephyra).

### 2.4. The Effect of ERK Molecular Knockdown on the Development of Metamorphosis

Ten polyps per hole were planted in a 24-well plate and starved one day before the experiment. Three siRNA sequences were designed for each molecule: si-Aco-evm.model.CTG_2.65_001 (CCCGAAATTTGATCACGCA), si-Aco-evm.model.CTG_2.65_002 (TGACATCAGTGACCCAAGT), and si-Aco-evm.model.CTG_2.65_003 (CCCTAACTTCCCTAAAGTA). The knockdown method referred to previous studies [66]. We set up one control group and three experimental groups with a total of 12 holes and 120 polyps. Preparation of the transfection complex was conducted by adding 2 μL of 20 μM siRNA storage solution (v3) to 1× riboFECT CP Buffer (v2); it was mixed gently. Subsequently, 12 μL riboFECT CP Reagent (v4) was added, gently blown, and mixed evenly; furthermore, it was incubated at a normal temperature for 15 min to prepare the transfection complex. The transfection complex was then added to a six-hole plate containing 1.866 mL artificial seawater (filtered once after mixing to remove impurities in the water). The hydra was cultured in the incubator for 48 h, and then the liquid was changed. Artificial seawater (2 mL), containing 50 μM 5-methoxy-2-methylindole, was added to the control group, and different siRNA solutions were added to the experimental group. The metamorphosis of polyps was observed every 24 h and recorded until the disk was released. The siRNA sequences were picked out with an obvious inhibitory effect, the above siRNA knockdown verification was repeated, the total RNA was extracted, and RT-QPCR verification was performed.

### 2.5. Extraction of Total RNA from the Samples

In our laboratory, the RNA extraction methods used for polyps are different from those applied to mammals owing to the high sugar and water contents of polyps [70]. Fifty polyps were placed into 2 mL RNase-free centrifuge tubes with 400 μL of SDS lysate (SDS extract + 10% β-mercaptoethanol). Grinding beads were added, and the tissue was ground for 60 s (every 20 s, the machine was stopped for 15 s, and the process was repeated 3 times). SDS lysate (300 μL) was then added, followed by 600 μL of saturated phenol chloroform isoamyl alcohol (25:24:1), and shaken on ice for 10 min. A total of 500 μL of supernatant was collected into a 1.5 mL RNase-free centrifuge tube, rapidly mixed with an equal volume of isopropanol (concussion for 15 s), and placed on ice for 15 min. Then, chloroform (200 μL) was added, and the tube was inverted violently for 15 s, placed on ice for 3 min, and centrifuged at 15,616× *g* and 4 °C for 15 min. A total of 500 μL of supernatant was transferred by a liquid transfer gun to a new 1.5 mL RNase-free tube. An equal volume of ice-cold isopropanol was added immediately, mixed evenly and quickly, and set aside for 8 min on ice. The mixture was centrifuged at 15,616× *g* and 4 °C for 10 min, then the supernatant was poured out. The precipitate was washed with 1 mL of 75% ethanol and centrifuged for 5 min at 4 °C and 5345× *g*. The supernatant was then discarded, and the wash step was repeated once. Then, the tube with the liquid was centrifuged at 5345× *g* for 1 min at 4 °C and the alcohol was drawn away with an RNase-free gun tip. DEPC-treated water (25 μL) was used to resuspend the RNA. RNA degradation and contamination were assessed by 1% agarose gel electrophoresis. RNA purity and concentration were measured using a Qubit 2.0 Fluorometer.

### 2.6. RT-qPCR

According to the instructions, REverTra qPCR RT Master Mix was reverse-transcribed into cDNA, and real-time quantitative polymerase chain reaction was used to detect the expression of the target gene. The target gene (ERK molecular evm.model.CTG_2.65) was provided by Jin Weizhi Company. The GAPDH gene was used as an internal control, and the primer sequences of the target gene and reference gene are listed in Table 1. The total reverse transcription system was 20 μL, and the RNA required was 1 μg. According to the determined RNA concentration, 1000/CRNA is the amount of RNA added, and the rest is filled with DEPC water. The reverse transcription conditions were as follows: 37 °C for 15 min, 50 °C for 5 min, 98 °C for 5 min, and cooling at 4 °C for 20 min. After reverse transcription, 20 μL of cDNA was removed and placed in a 1.5 mL enzyme-free EP tube, and 480 μL of DEPC water was added and mixed well. The primers were prepared with DEPC water. According to the instructions, we chose the PCR amplification system of 20 μL:1 μL R-terminal primers, 1 μL F-terminal primers, 10 μL SYBR, and 8 μL cDNA. The reaction conditions were as follows: pre-denaturation at 94 °C for 5 min, followed by 40 cycles of denaturation for 20 s at 94 °C, annealing for 20 s at 60 °C, and extension for 30 s at 72 °C. The detection was carried out on an Eppendorf MasterCycler realplex2 (Eppendorf). All experiments were repeated at least three times. The level of gene expression was calculated using the ^ΔΔ^C(t) method [71].

### 2.7. Quality Control, De Novo Assembly, and Functional Annotation

We collected some polyps and early strobila and washed them with artificial seawater; each group consisted of 4 centrifuge tubes with a volume of 2 mL, each tube of samples could be used to extract RNA, and there were 30 samples in each tube. After obtaining the total RNA, the cDNA library was constructed from the extracted RNA by technology, the Illumina NovaseqTM 6000 sequence platform was used to sequence the obtained paired readings, and CutAdapt was used to process the raw data from the previous machine for removal to obtain high-quality data. We used the HISAT2 (version: hisat2-2.2.1) software package to compare the readings of all samples with the reference genome of the research species (https://daehwankimlab.github.io/hisat2/, version:hisat2-2.2.1, accessed on 12 September 2021). The software package compares all readings to the reference genome, and for downstream analysis, FASTQC (https://www.bioinformatics.babraham.ac.uk/projects/fastqc/, accessed on 12 August 2024) was used to evaluate the quality obtained. We used StringTie 2.1.6 (http://ccb.jhu.edu/software/stringtie/, accessed on 12 August 2024) with default parameters to assemble the map readings for each sample. The FPKM value was calculated using StringTie and ballgown. The transcript of the Trinity is merged into the cluster according to the shared sequence content and can be regarded as a “gene”. The longest transcript in the same cluster is selected as the “gene” sequence (or “Unigene”). All the identified single genes were annotated in the GO (http://geneontology.org) and Jingdu Encyclopedia of Gene and Genome (KEGG) (https://www.kegg.jp/kegg/, accessed on 12 August 2024) databases, and their different fold changes (FCs) were more than 2 and *p* < 0.05. All the identified single genes were annotated in the database of Gene Ontology (http://geneontology.org) and Jingdu Encyclopedia of Gene and Genome (https://www.kegg.jp/kegg/, accessed on 12 August 2024). When log2 (FC) > 1 or log2 (FC) < −1, the R software package edgeR was used to select the differentially expressed genes according to statistical significance.

### 2.8. Statistical Analysis

The parameter was calculated as follows: Strobilation rate (SR, %) = strobilation number/total number × 100. Analyses were performed using GraphPad Prism 8.0.2 (GraphPad Software, 225 Franklin Street. Fl. 26. Boston, MA, USA) and SPSS program v27.0 (SPSS Inc., Michigan Avenue, Chicago, IL, USA) for Windows. All data are expressed as mean ± standard deviation (SD). The differences between groups were evaluated by a two-way ANOVA method, which considered time, concentration, and their interaction. For all tests, *p* < 0.05 was considered statistically significant. Additionally, alpha levels were corrected by Bonferroni for multiple comparisons.

## 3. Results

### 3.1. Homosalate Inhibits the Metamorphosis of Aurelia coerulea Polyps

**Figure 2:**
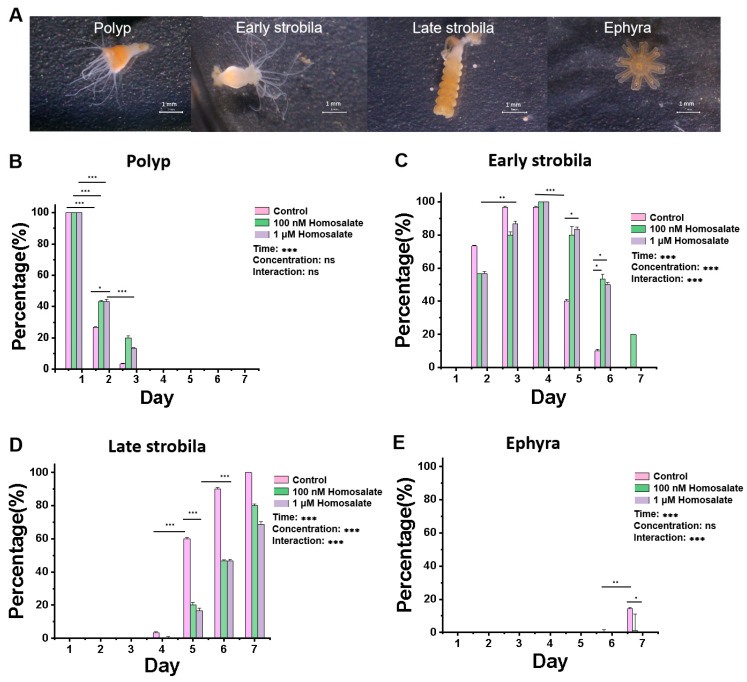
Homosalate inhibits the metamorphosis of *Aurelia coerulea* polyps. (**A**) The whole process of metamorphosis of *Aurelia coerulea* polyps under the somatotype microscope (scale bar: 1 mm). (**B**–**E**) The effect of 1 μM and 100 nM homosalate on the process of *Aurelia coerulea* metamorphosis in polyps, early strobila, late strobila and ephyra, respectively (**n** = 30, ^ns^ no significance, * *p* < 0.05, ** *p* < 0.01, *** *p* < 0.001).

We selected a chemical inducer configuration (5-methoxy-2-methylindole, salinity 2.6–3.2‰ artificial seawater, homosalate) with a final concentration of 100 nM and a 1 μM homosalate solution to treat polyps for 7 days. As shown in Figure 2, we analyzed the changes in each status of jellyfish over time and compared their differences between different groups. The results were as follows: In Figure 2B, *p* < 0.001 at the time level, *p =* 0.04 at the concentration level, and *p =* 0.061 at the interaction level. In Figure 2C, *p* < 0.001 at the time level, *p* < 0.001 at the concentration level, and *p* < 0.001 at the interaction level. Since *p* was less than 0.01 at the interaction level, we separately analyzed the differences between various concentrations at each treated time. On the fifth and sixth days, there were significant differences between the control group and different concentrations of homosalate, and *p* = 0.0332 existed between the control group and 1 μM homosalate group. In Figure 2D,E, *p* < 0.001 at the time level, *p* < 0.001 at the concentration level, and *p* < 0.001 at the interaction level. Since *P* was less than 0.01 at the interaction level, we separately analyzed the differences between various concentrations at each treated time. On the fifth and sixth days, there were significant differences between the control group and homosalate group.

Compared with the control group, the two concentrations of homosalate solution delayed metamorphosis and reduced the strobilation rate of *Aurelia coerulea* on the second day. In the control group, early strobila appeared on the second day, late strobila appeared on the fourth day, and ephyra appeared on the seventh day; under the treatment of 1 μM and 100 nM homosalate, late strobila appeared on the fifth day, and ephyra appeared on the seventh day, or later. There was no significant difference in inhibitory effects between 1 μM homosalate and 100 nM homosalate (Figure 2).

### 3.2. YS-49 Inhibits the Metamorphosis of Aurelia coerulea Polyps

**Figure 3:**
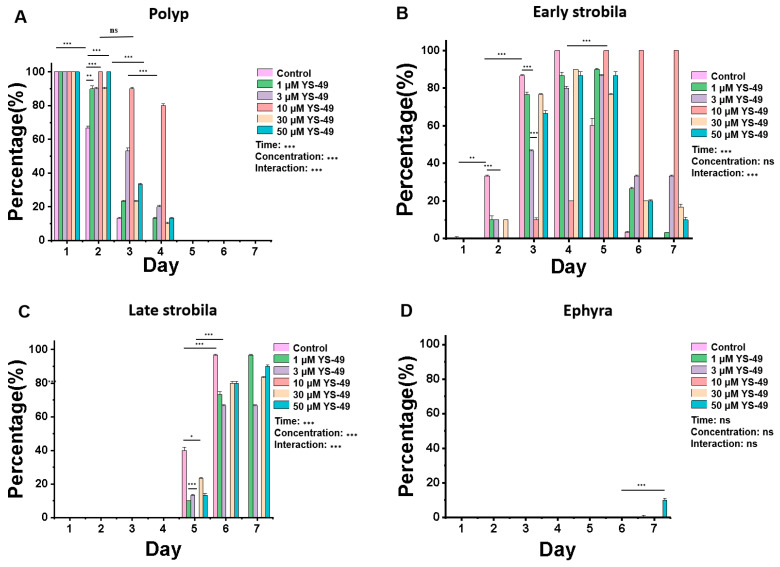
YS-49 inhibits the metamorphosis of *Aurelia coerulea* polyps. (**A**–**D**) The effect of 1 μM, 3 μM, 10 μM, 30 μM, and 50 μM YS-49 solution on the process of *Aurelia coerulea* metamorphosis in polyps, early strobila, late strobila, and ephyra, respectively (n = 30, ^ns^ no significance* *p* < 0.05, ** *p* < 0.01, *** *p* < 0.001).

YS-49 solutions with a final concentration of 1 μM, 3 μM, 10 μM, 30 μM, and 50 μM with a chemical inducer configuration (5-methoxy-2-methylindole, salinity 2.6–3.2, artificial seawater, and YS-49) were selected to treat polyps. As shown in Figure 3, we analyzed the changes in each status of jellyfish over time and compared their differences between different groups. The results were as follows: In Figure 3A, *p* < 0.001 at the time level, *p* < 0.001 at the concentration level, and *p* < 0.001 at the interaction level. Since *p* was less than 0.01 at the interaction level, we separately analyzed the differences between various concentrations at each treated time; on the second day, compared with the control group, all concentrations of YS-49 had significant effects. On the third and fourth days, compared with other groups, 10 μM YS-49 had significant differences. In Figure 3B, *p* < 0.001 at the time level, *p* = 0.248 at the concentration level, and *p* < 0.001 at the interaction level. Since *p* was less than 0.01 at the interaction level, we separately analyzed the differences between various concentrations at each treated time; on the second day, there were significant differences between the control group and different concentrations of homosalate. On the third day, compared with the control group, there were significant differences between 3 μM YS-49 and 10 μM YS-49. In Figure 3C, *p* < 0.001 at the time level, *p* < 0.001 at the concentration level, and *p* < 0.001 at the interaction level. Since *p* was less than 0.01 at the interaction level, we separately analyzed the differences between various concentrations at each treated time; on the fifth day, there were significant differences between the 10 μM YS-49 homosalate and other groups. In Figure 3D, *p* > 0.4 at the time level, concentration level, and interaction level. Additionally, in the control group, early strobila appeared on the 2nd day, late strobila appeared on the 5th day, and ephyra appeared on the 7th day; under five different concentrations of YS-49, no late strobila and ephyra appeared in the 10 μM YS-49 group on the 7th day. On the second day, the number of polyps in the control group was 20, and the percentage was 66.67%, while the number of polyps in 1 μM, 3 μM, 10 μM, 30 μM, and 50 μM YS-49 was 27, 27, 30, 27, and 30, respectively, and the percentage was 90%, 90%, 100%, 90%, and 100%, which was significantly higher than the control group. The results showed that, compared with the control group, the YS-49 solutions of five concentrations could delay the progress of metamorphosis and reduce the strobila rate of *Aurelia coerulea* from the second day. When compared with different doses of YS-49 in our study, the inhibitory effect of 10 μM YS-49 was the most significant, and 3 μM YS-49 was the second most significant (Figure 3).

### 3.3. Effect of ERK Molecular Knockdown on Development and Metamorphosis of Aurelia coerulea Polyps

**Figure 4:**
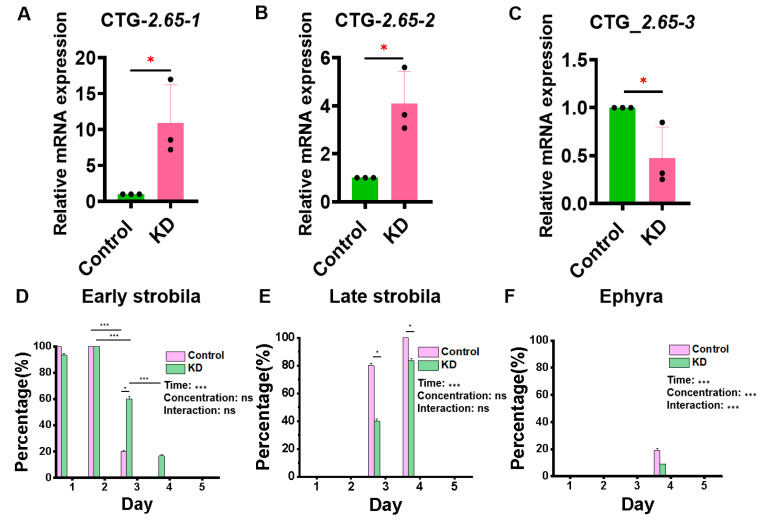
Knocking down ERK inhibits the metamorphosis process of *Aurelia coerulea* polyps. (**A**–**C**) RT-qPCR verification result of ERK knockdown. (**D**–**F**) The effect of knocking down ERK (evm.model.CTG_2.65) on the process of *Aurelia coerulea* metamorphosis in early strobila, late strobila, and ephyra, respectively (n = 30, ^ns^ no significance, * *p* < 0.05, *** *p* < 0.001).

According to the analysis of transcriptome data, the most significant expression of the ERK molecule in polyps and early strobila was evm.model.CTG_2.65. The knockout of three bands of ERK molecules is shown in Figure 4A–C, indicating that evm.model.CTG_2.65-3 was knocked down successfully. Then, siRNA-3 was selected to knock down the ERK molecule, and the metamorphosis development process of the *Aurelia coerulea* polyps was observed. As shown in Figure 4D,E, both *p* values were less than 0.001 at the time level when we analyzed the changes in each status of jellyfish over time and compared their differences between different groups. On the 3rd day, 6 polyps in the control group entered the early strobila, 24 polyps entered the late strobila, the percentage of early strobila was 20%, and that of late strobila was 80%. In the experimental group, on the 3rd day, 9 polyps were in the early strobila, 21 entered the late strobila, the percentage of early strobila was 30%, and that of late strobila was 70%. On the 4th day, all 30 polyps in the control group entered the late strobila, and the late strobila produced seven ephyra; the percentage of late strobila was 81.08%, and that of ephyra was 18.92%. In the experimental group, all 30 polyps had entered the late strobila, three ephyra were produced, the percentage of late strobila was 90.91%, and that of ephyra was 9.09%, which indicates that the development and metamorphosis of *Aurelia coerulea* polyps could be significantly delayed by ERK knockdown.

### 3.4. Signaling Pathway Map of Metamorphosis of Aurelia coerulea Polyps

**Figure 5:**
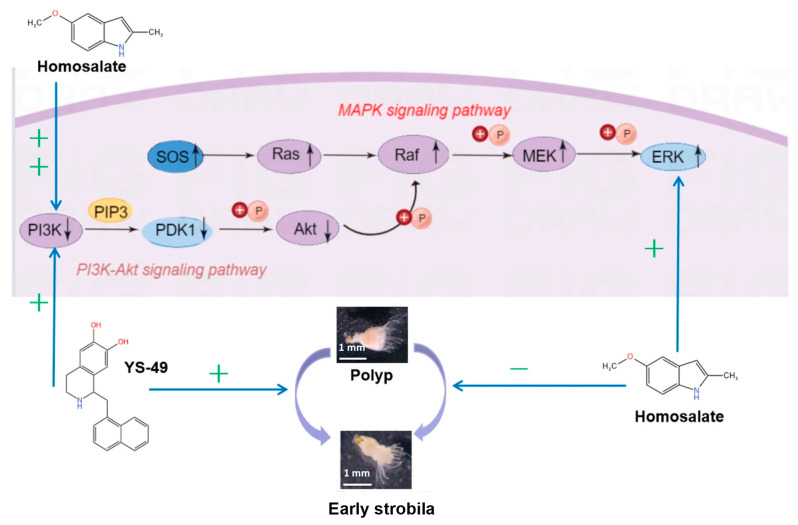
Signal pathway map of metamorphosis of *Aurelia coerulea* polyps (scale bar: 1 mm. → means function, + means activate, − means prohibit).

The expressions of PI3K, PDK1, and Akt, key molecules in PI3K-Akt signaling [72,73], which plays a negative regulatory role in the MAPK classical pathway, were all down-regulated, suggesting that environmental changes may simultaneously activate classical MAPK and inhibit PI3K/Akt signaling. We observed that homosalate activates both PI3K and ERK molecules [74]. YS-49 [75] activates the PI3K/Akt pathway, while ERK serves as a common molecule in both the PI3K/Akt and MAPK signal pathways. Treatment of *Aurelia coerulea* polyps with homosalate and YS-49 resulted in delayed metamorphic development. Conversely, when ERK (evm.model.CTG_2.65) was knocked down, a similar postponement in the metamorphic development of *Aurelia coerulea* polyps was observed. Our experimental findings from the two agonists and ERK knockdown were synthesized into a schematic diagram illustrating the mechanism by which agonists regulate the metamorphic development of *Aurelia coerulea* polyps through signaling pathway molecules (Figure 5). Simultaneous ERK inhibition and PI3K activation were associated with delayed metamorphosis in *Aurelia coerulea* polyps. The observed delay in metamorphosis induced by homosalate is suggested to be attributed to a stronger activation of PI3K relative to ERK.

## 4. Discussion

### 4.1. Control of Jellyfish Outbreaks from the Perspective of Regulating Metamorphic Development

Jellyfish (Cnidaria) are abundant and important members of many marine habitats, and their life cycle consists of three main stages, with the jellyfish stage having the greatest ecological and human impact [22]. Frequent jellyfish outbreaks constitute a global problem. There are approximately 500,000 jellyfish stings in Chesapeake Bay in the United States alone every year [39]. Serious jellyfish incidents can result in rapid fatality within a brief time frame. Moreover, jellyfish blooms can devastate the fishing and tourism industries, amounting to hundreds of millions of dollars in economic losses annually while degrading the livelihoods of coastal communities [76]. Such trends underscore the pressing need for a deeper understanding of the factors driving jellyfish proliferation, particularly in light of ongoing environmental changes and increased anthropogenic influences. 

### 4.2. Signaling Pathways in Jellyfish Metamorphosis

The metamorphosis of jellyfish, especially in *Aurelia coerulea*, is a complex process regulated by intricate signaling pathways. In our study, we focused on the roles of the MAPK and PI3K/Akt pathways, both critical for cellular processes such as proliferation, differentiation, and regeneration. The Wnt signaling pathway, known for orchestrating body axis formation, and the Hippo pathway, which regulates cell growth and tissue homeostasis, are also integral in jellyfish development [42,43]. Specifically, the ERK branch of the MAPK pathway is responsible for driving critical processes including cell proliferation and differentiation, alongside facilitating regeneration in hydroid species [44]. Additionally, specific inhibitors of PI3K can inhibit bud formation in hydra [45]. The Notch target genes are necessary for budding in hydra [46]. Homosalate, as an ultraviolet-blocking agent, is added to numerous skincare products that accumulate in aquatic ecosystems and human bodies, harming the marine ecological environment and human health. Furthermore, homosalate can activate PI3K and ERK simultaneously. However, reports on the effect of homosalate on the metamorphosis of *Aurelia coerulea* polyps are lacking. In this study, we found that the metamorphosis of *Aurelia coerulea* polyps was delayed when homosalate solutions of 100 nM and 1 μM were applied. ERK interacts with PI3K signaling and participates in cell proliferation and cytodifferentiation [74]. Additionally, the MAPK signaling pathway can down-regulate the SOS level and weaken PI3K/Akt signaling pathway transduction [77]. Reviews and histological data show that PI3K/Akt signaling regulates Drosophila metamorphosis [78,79,80] and participates in the process of embryonic diapause [81], while the transmission of the MAPK signal pathway changes gene expression and induces morphological changes in nematodes [82]. Given its involvement in the development of Drosophila, it can be assumed that the PI3K/Akt and MAPK signaling pathways regulate the growth and development of the body.

In this experiment, we observed that YS-49 inhibits the metamorphosis of *Aurelia coerulea* polyps by activating PI3K/Akt while knocking down ERK delayed development, which indicates that the activation of ERK may promote the metamorphosis process. Homosalate, a PI3K agonist and an ERK agonist, also delayed the jellyfish metamorphosis process, although its effect was less than YS-49, a specific PI3K agonist. All of those indicated that the activation of the PI3K/Akt pathway and inhibition of the ERK pathway play a significant inhibitory role in the metamorphosis process of jellyfish.

### 4.3. Implications for Environmental Management and Conservation

The escalating levels of homosalate and similar pollutants in marine ecosystems present a growing concern for jellyfish populations and, by extension, marine health. These invertebrates, serving as a novel toxicity experiment model [83,84,85], interact extensively with various chemicals due to their prolonged marine exposure. Concurrently, global jellyfish outbreaks have intensified in recent years, leading to substantial repercussions for ports, nuclear power plants, tourism, and medical sectors [17,86,87], The metamorphosis of jellyfish significantly contributes to their proliferation, yet effective interventions to mitigate outbreaks are lacking. The regulatory mechanisms governing jellyfish development remain unclear, and the treatment for jellyfish stings remains symptomatic with no definitive cure. The findings from this study reveal the inherent vulnerability of *Aurelia coerulea* polyps to environmental stressors, presenting a compelling case for the regulation of pollutants like homosalate to safeguard marine species and ecosystems. Furthermore, addressing the ineffective measures currently dominant in jellyfish sting mitigation—largely symptomatic—could lead to the development of targeted interventions that incorporate an understanding of the molecular pathways affected by environmental pollutants. Ultimately, our research elucidates critical regulatory mechanisms in jellyfish development, opening pathways for further exploration of evolutionary adaptations and informing conservation efforts aimed at safeguarding marine biodiversity.

## 5. Conclusions

Homosalate activates PI3K and ERK simultaneously to delay the process of metamorphosis of *Aurelia coerulea*, and YS-49 inhibits the metamorphosis of *Aurelia coerulea* by activating PI3K particularly. Additionally, after ERK (evm.model.CTG_2.65) was knocked down, the process of metamorphosis of *Aurelia coerulea* also slowed down. These results indicate that activating PI3K and knocking down the expression of ERK (evm.model.CTG_2.65) play important roles in delaying the process of metamorphosis of *Aurelia coerulea*. Both PI3K pathway and ERK pathway intervention may be potential strategies for the prevention and control of jellyfish blooms.

## Figures and Tables

**Table 1:** Primer sequences of target gene and reference gene.

Number	Query ID	Forward	Reverse
A	evm.model.CTG_2.65	ACCTCACAGAACTTGTATCCACTCG	CGCATCCACTACTCCAAACATCAAC
B	GAPGH	CCGTGTTCCAGTCCCAGATGTTTC	CCTTGCTCTCTGATGCTGCCTTC

## Data Availability

The datasets generated and/or analyzed during the current study here are available from the corresponding author upon reasonable request.

## Appendix A

The full protocol of feeding polyps with shrimp eggs (Artemia) is divided into 4 steps as follows:

1. Prepare artificial seawater. Weigh 0.5 kg of sea salt, evenly dissolve it in 5 L water, stir with a glass rod, wait for 15 min, and use a salinometer to test the salinity as 2.8–3.2‰. If the salinity is outside this range, increase the amount of water or sea salt based on the measurement results to adjust the salinity within the required range.
2. Artemia incubation. Pour 2 g (a full spoon) of Artemia, and add 800 mL artificial seawater into the automatic beaker with the oxygen pumps. In summer, it takes 12 h to incubate fully, and 24–48 h in winter. After incubation, turn off the inflation pumps, wait for 10 min until there is obvious stratification (the top is brown shrimp shells, the middle is transparent seawater, and the bottom is orange Artemia), then pour out the floating shrimp shells on the upper layer quickly, and the orange particles at the bottom are the incubated Artemia. Collect the Artemia in a beaker and fill it with seawater again, wait for 10 min until the shrimp shells float and the Artemia sink, and then, pour out the floating shrimp shells quickly. The fresh Artemia is stored in a refrigerator at 4 °C, and should be used up within 2 days after marking the date.
3. Feeding. Turn off the oxygen pumps and filtration devices in the large water tank for raising polyps. It is necessary to ensure that the Artemia is fresh, and feed it directly to the tentacles of the polyps with a disposable Pasteur pipette so that each polyp can eat fresh Artemia. After 2 h, the polyps will be fed, and it is time to change the water in the water tank for artificial seawater; then, the oxygen pumps are turned on. They are fed every two days.
4. Environmental control of the water tank. In order to ensure that the salinity and specific gravity are within the normal range, water should be added to the water tank every day to replenish the evaporated seawater; the filter cotton should be cleaned every two days; the water should be changed thoroughly and the tank wall and bottom cleared every week.
